# Clerihews

**Published:** 1983

**Authors:** 


### CLERIHEWS

**PRESIDENTIAL NOTES**

1980–1981

Dr. Norman Brown,
Autopsist of renown,
Per (every working) diem
Has carried out a PM.

1981–1982

Dr. Robert Warin
Will gladly let you share in
His knowledge of the hide,
Which is now extremely wide.

1982–1983

Mr. Herbert Bourns
Will not cut your corns,
But he will use his knife
To save your life.

M.G.W. after E.C.B.

